# Protein Carbonylation in Patients with Myelodysplastic Syndrome: An Opportunity for Deferasirox Therapy

**DOI:** 10.3390/antiox8110508

**Published:** 2019-10-24

**Authors:** Alba Rodríguez-García, María Luz Morales, Vanesa Garrido-García, Irene García-Baquero, Alejandra Leivas, Gonzalo Carreño-Tarragona, Ricardo Sánchez, Alicia Arenas, Teresa Cedena, Rosa María Ayala, José M. Bautista, Joaquín Martínez-López, María Linares

**Affiliations:** 1 Department of Hematology, 16473 Hospital Universitario 12 de Octubre, Hematological Malignancies Clinical Research Unit H120-CNIO, 28041 Madrid, Spain; albarodriguezgarcia@hotmail.com (A.R.-G.); mlmorales17@gmail.com (M.L.M.); gvaneg@hotmail.com (V.G.-G.); irene.g.baquero@gmail.com (I.G.-B.); alejandraleial@gmail.com (A.L.); gonzalocarreno.gomeztarragona@gmail.com (G.C.-T.); ricardsanchez.hdoc@gmail.com (R.S.); arenasalic@gmail.com (A.A.); mariateresa.cedena@salud.madrid.org (T.C.); rayaladiaz12@gmail.com (R.M.A.); mlinares@ucm.es (M.L.); 2 Department of Biochemistry and Molecular Biology and Research Institute Hospital 12 de Octubre, Universidad Complutense de Madrid, Ciudad Universitaria, 28040 Madrid, Spain; jmbau@vet.ucm.es; 3 Department of Medicine, Universidad Complutense de Madrid, Ciudad Universitaria, 28040 Madrid, Spain

**Keywords:** myelodysplastic syndromes, carbonylation, oxidative stress, deferasirox

## Abstract

Control of oxidative stress in the bone marrow (BM) is key for maintaining the interplay between self-renewal, proliferation, and differentiation of hematopoietic cells. Breakdown of this regulation can lead to diseases characterized by BM failure such as the myelodysplastic syndromes (MDS). To better understand the role of oxidative stress in MDS development, we compared protein carbonylation as an indicator of oxidative stress in the BM of patients with MDS and control subjects, and also patients with MDS under treatment with the iron chelator deferasirox (DFX). As expected, differences in the pattern of protein carbonylation were observed in BM samples between MDS patients and controls, with an increase in protein carbonylation in the former. Strikingly, patients under DFX treatment had lower levels of protein carbonylation in BM with respect to untreated patients. Proteomic analysis identified four proteins with high carbonylation levels in MDS BM cells. Finally, as oxidative stress-related signaling pathways can modulate the cell cycle through p53, we analyzed the expression of the p53 target gene p21 in BM cells, finding that it was significantly upregulated in patients with MDS and was significantly downregulated after DFX treatment. Overall, our results suggest that the fine-tuning of oxidative stress levels in the BM of patients with MDS might control malignant progression.

## 1. Introduction

Myelodysplastic syndromes (MDS) are a heterogeneous group of hematopoietic stem cell disorders characterized by the presence of immature myeloid precursors (blasts) and dysplastic hematopoiesis in the bone marrow (BM). A current “hot” topic in MDS research is oxidative stress and its potential effects on cell biology, DNA damage and carcinogenesis [1,2]. Whereas the origin of MDS disease is now better understood, high levels of reactive oxygen species (ROS) and consequent oxidative damage in hematopoietic cells have been reported in patients with this disease [3,4,5,6], but the consequences are less clear. Regulation of intracellular ROS levels is known to be key for maintaining the balance between self-renewal, proliferation, and differentiation of progenitor cells and a loss of this control can lead to diseases characterized by BM failure [7,8]. The potential effects of ROS on hematopoietic cells are particularly relevant because they are acutely vulnerable to oxidative damage associated with the accumulation of free radicals [9].

Studies have shown that BM cells from patients with MDS have increased levels of intracellular peroxide and decreased levels of the antioxidant glutathione (GSH), as compared with normal cells [10,11,12]. Importantly, patients with MDS and with high ROS or low GSH levels, and a high superoxide/peroxide ratio, have a lower overall survival [10]. The effects of increased intracellular ROS production are well recognized and include direct damage to biomolecules and/or dysregulation of ROS-dependent signaling pathways [8,13,14]. In the context of blood cells, an interplay has been reported between oxidative damage of DNA in CD34+ cells and subsequent increased oxidation levels in precursor cells such as blasts or erythroid precursors [4]. Also, oxidative stress correlates with DNA hypermethylation in patients with MDS [11] and other pathological conditions [15].

Proteins are important targets of ROS [16], and oxidation can lead to aggregation, polymerization, unfolding or conformational changes that cause structural or functional loss. Although several oxidative modifications to proteins are possible, most involve the formation of carbonyl groups, which are irreversibly introduced into proteins [17,18,19]. Protein carbonylation can occur by several pathways, but the two main contributors are: (i) direct metal-catalyzed oxidation of specific amino acid residues (lysine, arginine, proline and threonine), and (ii) secondary reactions of nucleophilic amino acid side-chains with ROS-induced lipid peroxidation products such as 4-hydroxynonenal (HNE) [17,19]. These modifications likely have important roles in cell signaling [18,20].

As a specific marker of oxidative damage, assessing protein carbonylation is useful for understanding the role of oxidative stress in several disease [19]. A common method of protein carbonylation detection involves derivatization by 2, 4-dinitrophenylhydrazine (DNPH) to form a stable dinitrophenylhydrazone (DNP) product, which is subsequently detected by specific antibodies to visualize carbonyl groups bound to tissues and cells. This method can therefore provide information both on the distribution of in vivo oxidative damage, and the identification of their protein targets by complementary methods such as mass spectrometry (MS) [18,21,22]. 

Signaling pathways activated by oxidative stress can control the cell cycle via the p53 gene [23]. Indeed, the p53 target gene p21, a cyclin-dependent kinase inhibitor, is involved in the cell response to genotoxic stress [16] and has a role in the regulation of senescence by controlling proliferation [24]. Defective activation of p21 can lead to leukemogenesis [25,26]. Along this line, it has been suggested that during the first phase of MDS, the ineffective maturation of progenitor hematopoietic cells is associated with an increase in apoptosis, whereas in more advanced stages, BM cells switch to a proliferative phenotype with negligible apoptotic control [27]. 

Many patients with MDS require frequent blood transfusions and can develop transfusion-dependent iron-overload [28], which in turn can lead to an increase in the generation of ROS [29]. Accordingly, iron chelator therapy has been proposed to address primary oxidative stress in MDS [28,30,31]. Indeed, Neukirchen et al. [32] presented data showing a survival benefit for low-risk MDS patients receiving iron chelation therapy. One such treatment related to oxidative stress that has been used in patients with MDS is the iron chelator deferasirox (DFX) [33]. DFX seems to constrain ROS damage by activating transcription factors and mitochondrial biogenesis [34], and by inhibiting the *de novo* generation of free radicals through the suppression of the active redox forms of iron [35]. Protein carbonylation can be reduced/eliminated with DFX, which can trap Fe-III ions by forming a 1:2 octahedral complex and preventing their reduction [36]. 

In this report, we demonstrate for the first time the therapeutic benefit of DFX to inhibit protein carbonylation associated with MDS. Moreover, we provide direct evidence of four proteins with increased carbonylation in the BM of patients. Lastly, our results suggest that the oxidative damage in MDS is related, at least in part, to signaling pathways regulating the cell cycle through p53/p21, which are moderated by DFX treatment, overall highlighting the important role of DFX in the control of the oxidative stress response in MDS. 

## 2. Materials and Methods

BM samples were obtained from patients diagnosed with MDS (*n* = 21, median age 75 years; range 57–90 years) who were grouped according to the World Health Organization classification of tumors of hematopoietic and lymphoid tissues (2008), and to the International Prognostic Scoring System [37]. The main characteristics of the patients are summarized in Table 1. 

The control group consisted of individuals (*n* = 13, median age 69 years; range 29–94 years) with no signs of the disease in BM no infiltration of the disease in BM. The study was approved by the Comité Ético de Investigación Clínica of the Instituto de Investigación Biomédica of the Hospital 12 de Octubre, and all patients signed an informed consent after having understood all issues involved in the study participation, in accordance with the guidelines described in the Declaration of Helsinki, Convention of the Council of Europe on Human Rights and Biomedicine, Universal Declaration of the United Nations Educational, Scientific and Cultural Organization on the human genome and human rights and requirements established in Spanish legislation in the field of biomedical research, the protection of personal data and bioethics. 

Immunohistochemistry analysis was performed in MDS (*n* = 9), control (*n* = 7) and DFX-treated (*n* = 6) samples, as previously described [19], with some modifications. BM smears, previously fixed in methanol, were refreshed in phosphate buffered saline/1% bovine serum albumin, and endogenous peroxidase was inactivated by incubation for 5 min with 3% hydrogen peroxide. Antigen unmasking and recovery was performed by heat-mediated antigen retrieval with citrate buffer. Protein carbonylation was detected by sample derivatization with DNPH (ref. 04732) and specific detection of the DNP derivatives by indirect peroxidase staining [19] using an anti-DNP rabbit antibody (1/200 dilution; ref. D9656) (both from Sigma-Aldrich, St. Louis, MO, USA). An immunohistochemistry assay using an anti-4-HNE rabbit antibody (1:100 dilution, ref. ab46545) (Abcam, Cambridge, UK) was also performed. Smears were counterstained with Carazzi’s hematoxylin solution. Finally, samples were dehydrated in an ethanol series (100%, 96%), cleared in xylol and coverslipped with DPX mounting medium (Sigma-Aldrich). Samples were visualized and imaged using a Nikon Eclipse 80i microscope (Nikon, Tokyo, Japan) equipped with a Nikon digital camera. All cells were counted from 4 randomly chosen fields and averaged. 

**Figure A1:**
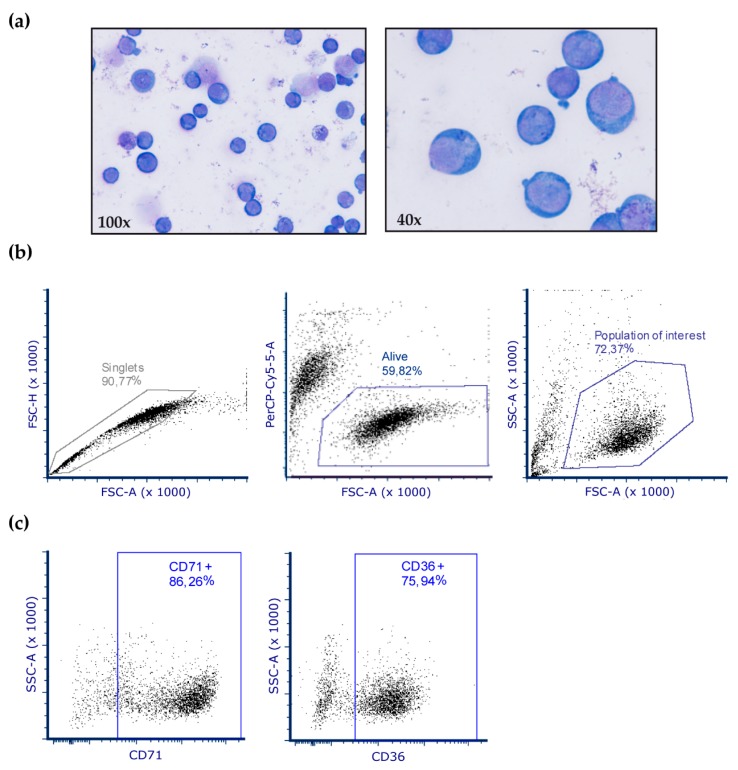
Characterization of erythroblasts in cell culture. (**a**) After 10 days of culture, cells were stained with Wright´s stain and 98% of cells were identified as erythroblasts. (**b**–**c**) Representative dot plots from flow cytometry analysis of erythroblast cultures. (**b**) Analysis and gating strategy of erythroblast cells. (**c**) Cells were analyzed after 10 days in culture for CD71 and CD36 expression, which is specific for erythroblasts.

Primary cultures used for OxyBlot assays were obtained from BM samples of patients with MDS (*n* = 8) and control subjects (*n* = 6). Mononuclear cells were separated by density gradient centrifugation through Ficoll (GE Healthcare, Chicago, IL, USA). Subsequently, CD34+ cells were selected using the CD34+ Microbead Kit and magnetic cell separation (MiniMACS, Miltenyi Biotec, Bergisch Gladbach, Germany). As previously described [38], cells were cultured in enriched Stem Spam medium with erythropoietin (0.5 U/mL, which was increased after six days), stem cell factor (100 ng/mL), IL-3 (10 ng/mL), lipoproteins (40 μg/mL), dexamethasone (1 μM), glutamine (2 mM) and 1% penicillin-streptomycin, with the aim of promoting erythroid precursor development. All cell culture products were purchased from Stem Cell Technologies (Vancouver, BC, Canada) with the exception of lipoproteins (Sigma-Aldrich, St. Louis, MO, USA). To characterize erythroblasts, after 10 days of culture, cells were stained with Wright’s stain and analyzed by flow cytometry on a BD FacsAria Fusion cytometer (BD Biosciences, Franklin Lakes, NJ, USA). The gating strategy was based on dead/live cells and doublet discrimination. When possible, a minimum of 10,000 events of the population of interest was analyzed. The erythroblast antigen expression profile was evaluated by labeling with the fluorescently-conjugated antibodies CD71-APC and CD36-PE. FCS Express 6 (De NovoSoftware, Glendale, CA, USA) was used for data analysis (Figure A1).

After 10 days in culture, cells were lysed in a buffer containing 50 mM Tris pH 8, 50 mM NaCl, 3% (*w*/*v*) CHAPS and protease inhibitors. Total protein lysates were denatured at 100 °C for 3 min in the presence of 6% sodium dodecyl sulfate (SDS) and then derivatized as previously described [18], with some modifications. One volume of a solution containing 10 mM DNPH in 10% trifluoroacetic acid was added to the samples followed by incubation for 10 min at 25 °C. The mixture was neutralized by adding one volume of stop solution (2 M Tris, 30% glycerol and 15% β-mercaptoethanol). Samples were precipitated and resuspended in a buffer containing 7 M urea, 2 M thiourea, 3% (*w*/*v*) CHAPS, 0.5% (*w*/*v*) MEGA-10, 0.5% (*w*/*v*) LPC, and 10 mM dithioerythritol. Protein quantification was performed with the Bradford 1× Dye Reagent Quick Start ™ reagent from Bio-Rad (Hercules, CA, USA) and the absorbance at 595 nm was read on an EPOCH plate reader (BioTek, Highland Park, VT, USA) using Gen5 2.0 software (BioTek).

After quantification, a one-dimensional (1D)-OxyBlot was performed with 4 μg of protein samples electrophoresed through a denaturing 10% SDS-polyacrylamide gel and transferred to polyvinylidene fluoride (PVDF) membranes. Membranes were incubated overnight at 4 °C with an anti-DNP primary antibody (as in immunohistochemistry assay, but at 1/10,000 dilution) followed by an anti-rabbit-IgG HRP-linked secondary antibody (1/5000 dilution; ref. 7074, Cell Signaling Technologies, Danvers, MA, USA). Glyceraldehyde-3-phosphate dehydrogenase (antibody Ref. AM4300, Ambion/ThermoFisher, Waltham, MA, USA) was used as a loading control. Chemiluminescense was used to visualize proteins according to the ECL Prime Western Kit Blotting Detection Reagent Amersham protocol from Sigma-Aldrich, on the ChemiDoc™ MP Imaging System (Bio-Rad). Images were captured and analyzed with the ImageLab 5.0 program (Bio-Rad).

For 2D-OxyBlot and preparative gel electrophoresis, four gels (*n* = 2 from MDS samples; *n* = 2 from control samples), with 100 μg protein per sample, were run in parallel under identical conditions and transferred to PVDF membranes. Membranes were incubated with anti-DNP primary and anti-rabbit-IgG HRP-linked secondary antibodies, as described for the 1D-OxyBlot. Chemiluminescense was performed as above with exposure on Curix RP2 PLUS film (AGFA, Mortsel, Belgium). Due to the limitation of available protein, an additional SDS-polyacrylamide gel containing a representative pool of the samples (400 μg) was run in parallel and stained with Colloidal Coomassie brilliant blue G250 to display total proteins and for subsequent protein identification by MS analysis.

Spots of interest were manually excised from the 2D preparative gel, in-gel reduced, alkylated, and then digested with trypsin, as described elsewhere [18]. Proteins were identified by matching the trypsin-digested peptide mass against the SwissProt database (SwissProt 553231 sequences; 197953409 residues) using MASCOT 1.9 (http://www.matrixscience.com) through the Global Protein Server (v3.5) from Applied Biosystems (Foster City, CA, USA). The parameters for the peptide mass fingerprinting search were as follows: modification on cysteine residues by carbamidomethylation was set as fixed modification; methionine oxidation was considered as a variable modification; the maximum number of missed tryptic cleavages was one; peptide mass tolerance was set to 50 ppm and monoisotopic masses were considered. In some cases, taxonomy was restricted to human. All of the identified proteins fulfilled the criterion of being significant (*P* < 0.05) based on the MOWSE scoring scheme. MS data have been deposited to the ProteomeXchange Consortium via the PRIDE partner repository [39], with the identifier code PXD013609. To corroborate that the signal level was due exclusively to a higher level of carbonylation and not to a higher protein expression, specific antibodies for the identified proteins were used.

Analysis of p21 mRNA expression was performed by quantitative PCR (qPCR). Synthesis of cDNA from BM-extracted RNA of MDS (*n* = 5), control (*n* = 8) and DFX-treated (*n* = 3) samples was performed with the High Capacity cDNA Reverse Transcription Kit (ThermoFisher) on the Veriti 96 Well Thermal Cycler platform (Applied Biosystems), maintaining a 1:1 ratio of reverse transcriptase buffer and RNA. Gene expression levels of p21 (ref. Hs00355782_m1) and the constitutive gene β-glucuronidase (Gus) (ref. Hs00939627_m1) gene were measured following the TaqMan^®^ Gene Expression Assay protocol provided by ThermoFisher. As a positive control, samples with known transcriptional p21 activation were used as a reference. All samples were tested in triplicate and the changes in gene expression were calculated using the comparative ΔCT method [40]. The Gus gene served as an endogenous control for any slight variation in the initial RNA concentration, the total RNA quality, and the conversion and the efficiency of the reverse transcription reaction. Finally, expression levels were normalized to control samples.

Data are presented as the mean ± SEM. Comparisons between two groups were performed using the parametric Student’s t-test or the non-parametric Mann-Whitney U test, as appropriate. Multiple group comparisons were performed using the parametric analysis of variance (ANOVA) or the non-parametric Kruskal-Wallis test. Differences were considered to be statistically significant at *P* ≤ 0.05. All analyses were performed using GraphPad Prism 5.01 software (GraphPad Software Inc., La Jolla, CA, USA).

## 3. Results

### 3.1. Protein Carbonylation is Increased in Myeloid Series from MDS Patients and is Decreased in Patients Treated with DFX

**Figure 1:**
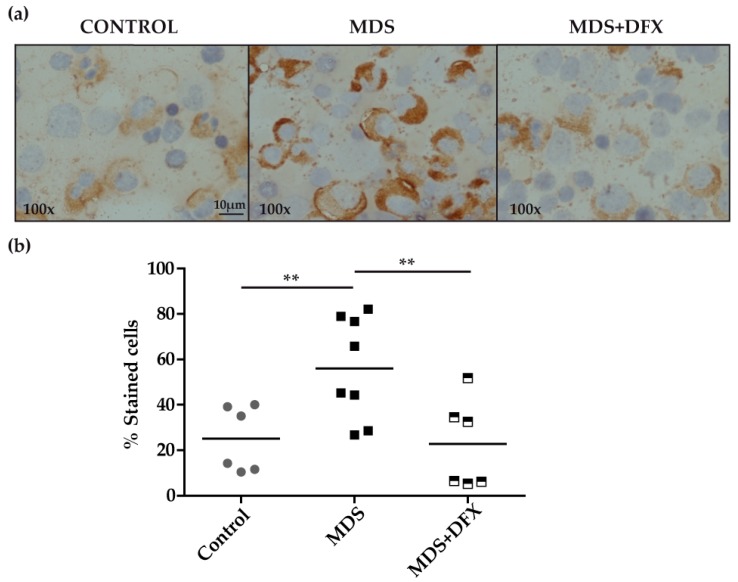
Immunodetection of carbonyl groups in derivatized bone marrow samples using an anti-DNP primary antibody. (**a**) Representative images (100× magnification) of bone marrow smears from controls and MDS patients treated or not with DFX (MDS and MDS+DFX, respectively), derivatized with 2,4-dinitrophenylhydrazine. (**b**) Quantification of carbonylation-positive cells detected by immunohistochemistry: *n* = 14: control (*n* = 6), MDS (*n* = 8), and MDS+DFX (*n* = 6); mean ± SEM, **P ≤ 0.01.

Immunohistochemistry analysis of BM samples revealed higher oxidative stress, measured by protein carbonylation, in MDS patients than in controls or in DFX-treated MDS patients (Figure 1a). Carbonylation immunostaining was mostly cytoplasmic and was evidently more intense in the myeloid series. Quantitative analysis, measured as the percentage of positive stained cells of different randomly chosen fields, showed that the number of positively stained cells was significantly higher (*P* ≤ 0.01) in MDS samples than in equivalent controls. In addition, BM samples from DFX-treated MDS patients had a significantly lower number of positively stained cells than untreated patients (*P* ≤ 0.01), whereas there was no statistically different change in the number of positive cells between samples from controls and DFX-treated MDS patients (*P* = 0.82) (Figure 1b). A negative control sample without primary antibody verified that staining was exclusively due to the presence of carbonyl groups. It should be noted that although DNPH is the most commonly used probe for this approach, it lacks the ability to characterize the type of carbonyl modification [17].

**Figure 2:**
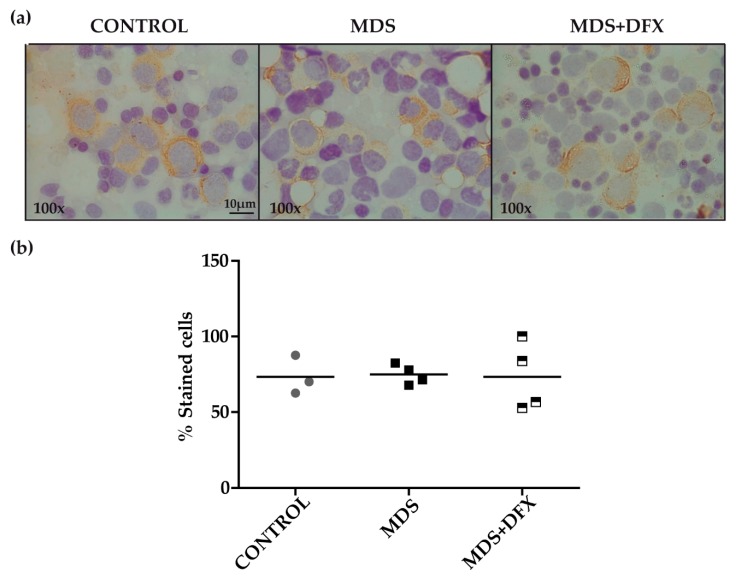
Immunodetection of 4-hydroxynonenal (HNE) in bone marrow samples. (**a**) Representative images (100× magnification) of bone marrow smears from controls, MDS and DFX-treated MDS patients (MDS+DFX), detected by anti 4-HNE immunostaining. (**b**) Quantification of 4-HNE-positive cells detected by immunohistochemistry: *n* = 11: control (*n* = 3), MDS (*n* = 4), and MDS+DFX (*n* = 4); mean ± SEM.

To examine for the presence of 4-HNE adducts, we performed an immunohistochemistry assay with MDS, control and MDS+DFX BM samples (Figure 2a). Quantitative analysis showed no statistically differences in the 4-HNE staining between these groups (*P* = 0.29) (Figure 2b).

### 3.2. Protein Carbonylation is Increased in MDS Erythroid Precursors and is Decreased by DFX Treatment

**Figure 3:**
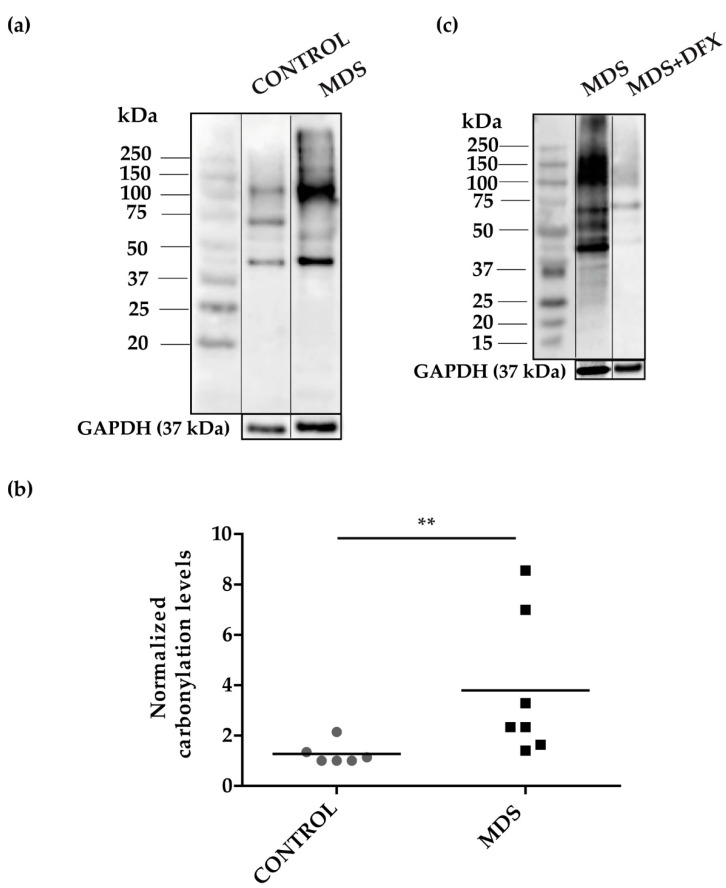
Protein carbonylation in erythroid precursor extracts. (**a**) Representative 1D-OxyBlot from cultured CD34+ cells of control (C) and MDS samples derivatized with DNPH and detected by anti-DNP immunostaining. (**b**) Quantification of normalized carbonylation levels by 1D-OxyBlots. *n* = 13: control (*n* = 6), and MDS (*n* = 7); mean ± SEM, ** *P* ≤ 0.01. (**c**) 1D-OxyBlot of protein extracts from cultured CD34+ cells from MDS and DFX-treated MDS BM samples. A protein standard is also shown for reference.

**Figure A2:**
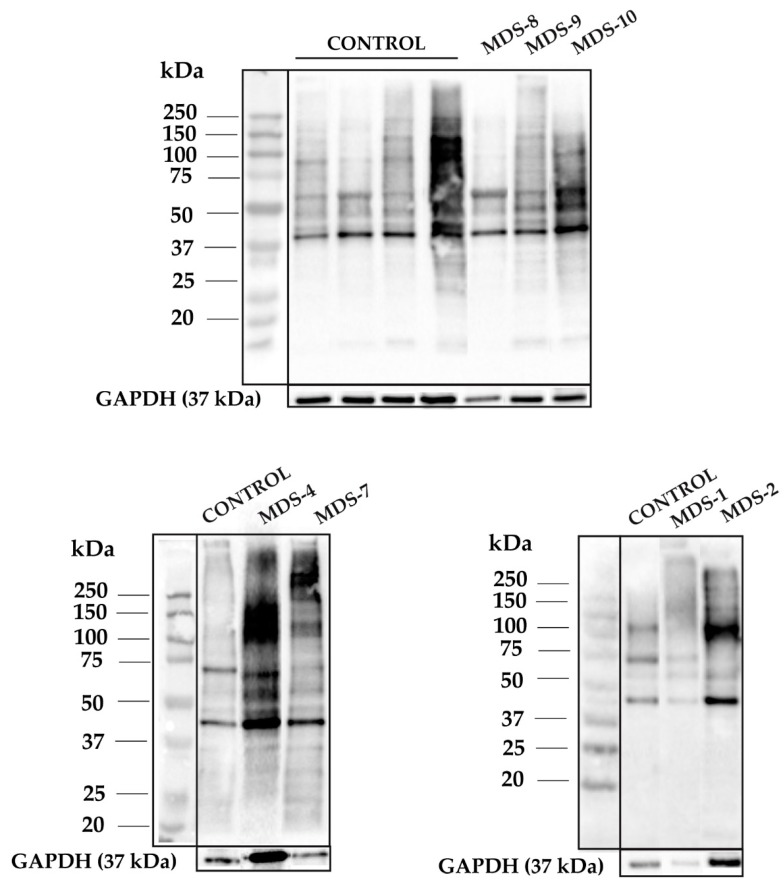
1D-OxyBlots from all cultured CD34+ cells of control and MDS samples derivatized with DNPH and detected by anti-DNP immunostaining. A protein standard is shown for reference.

**Figure A3:**
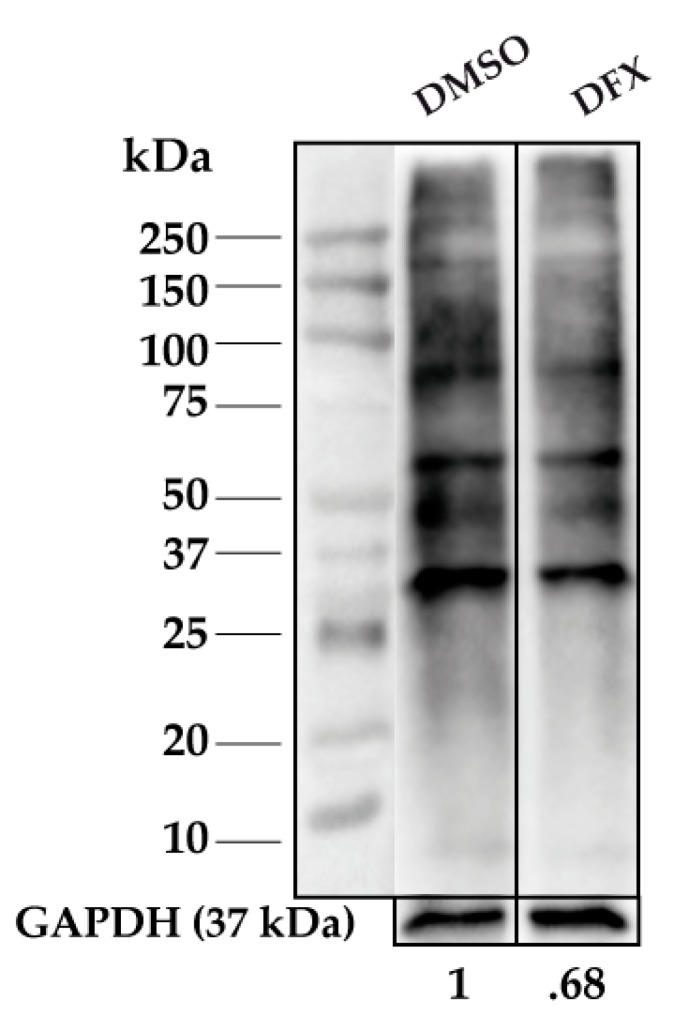
Representative 1D-OxyBlot from CD34+ cells treated with 50 μM DFX for 24 h, *n* = 2. Carbonylation levels were normalized to GAPDH levels (loading control).

To address the possibility of oxidative stress in MDS erythroid precursors, we examined the pattern of carbonylated proteins in cultured erythroblasts (Figure A1) from the three groups. We performed 1D-OxyBlot analysis of total protein lysates from CD34+ cells cultured from MDS and control BM samples. Results showed a significant increase (*P* ≤ 0.01) in protein carbonylation in MDS over control samples (Figure 3a,b and Figure A2). Closer inspection of the 1D-OxyBlots showed strongly staining carbonylated protein species in MDS erythroblasts ranging in molecular weight from 40 to 250 kDa, with a major band at 100 kDa. We also observed a particularly highly carbonylated ~40 kDa band present in all samples, which was stronger in MDS lysates (Figure 3a). As expected, we observed that carbonylated protein staining was considerably weaker in erythroblasts cultured from DFX-treated MDS BM (Figure 3c). Ex vivo treatment with DFX also produced a reduction in protein carbonylation (Figure A3).

**Figure 4:**
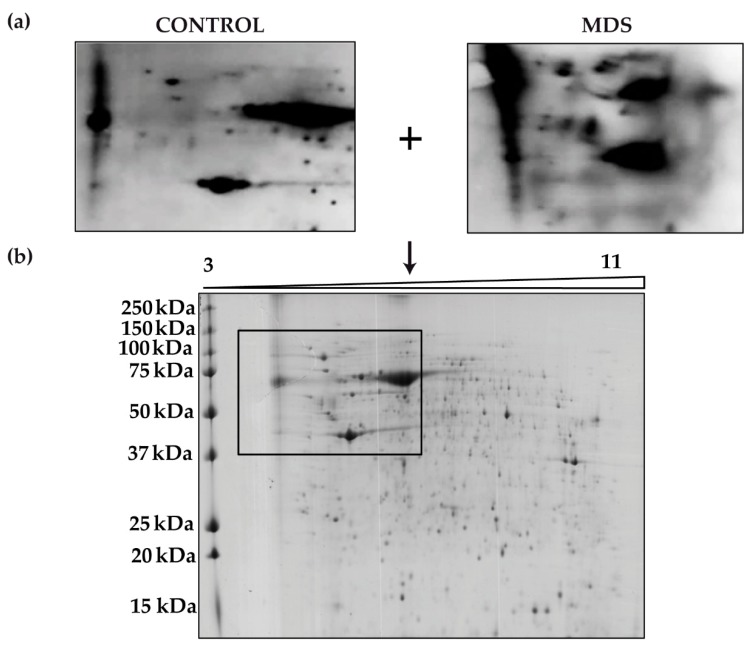
Protein carbonylation in erythroid precursors analyzed by 2D-Oxyblot (**a**) Representative 2D-OxyBlots of protein extracts from cultured CD34+ cells of representative control and MDS samples derivatized with DNPH and stained with an anti-DNP antibody. (**b**) Preparative 2D gel from pooled samples derivatized with DNPH and detected by Coomassie staining.

We next aimed to identify the proteins responsible for the overall increased carbonylation in MDS erythroblasts. To do this, we performed 2D-OxyBlot analysis (Figure 4a) of the carbonylated proteins in cultured erythroblasts. A clear difference in the pattern of carbonylated proteins could be observed in the 2D-OxyBlots between MDS and control samples (Figure 4a). Although a greater number of carbonylated proteins in MDS samples than in controls might be expected, only a small number of proteins appeared to be responsible for the increased levels of total protein carbonylation. Consistent with the results from 1D- and 2D-OxyBlots, proteins ranging in size from 37 to 250 kDa had the highest carbonylation levels (Figure 4b).

Four proteins with higher carbonylation levels in the MDS samples were excised from the preparative gel. MS analysis identified the proteins as: cytoplasmic actin, zinc finger protein 846 (ZNF846), 14-3-3 protein zeta/delta, and L-lactate dehydrogenase (LDH) A chain (Table 2). These four carbonylated proteins in MDS cells are related mainly to cell cytoskeleton, transcriptional regulation and metabolism.

### 3.3. Upregulation of p21 in MDS is Controlled by DFX

**Figure 5:**
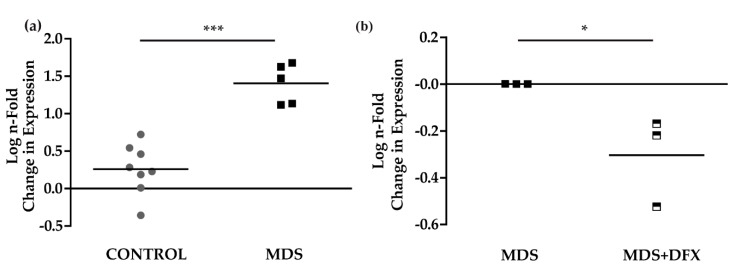
p21 mRNA expression. (**a**) Comparison of normalized p21 mRNA levels between samples from controls (C) (*n* = 8) and MDS patients (MDS) (*n* = 5); mean ± SEM ***P ≤ 0.001. (**b**) p21 mRNA levels from MDS patients treated with DFX (MDS+DFX) (*n* = 3) normalized to p21 levels before DFX treatment (MDS) (*n* = 3); mean ± SEM, *P ≤ 0.05.

**Figure A4:**
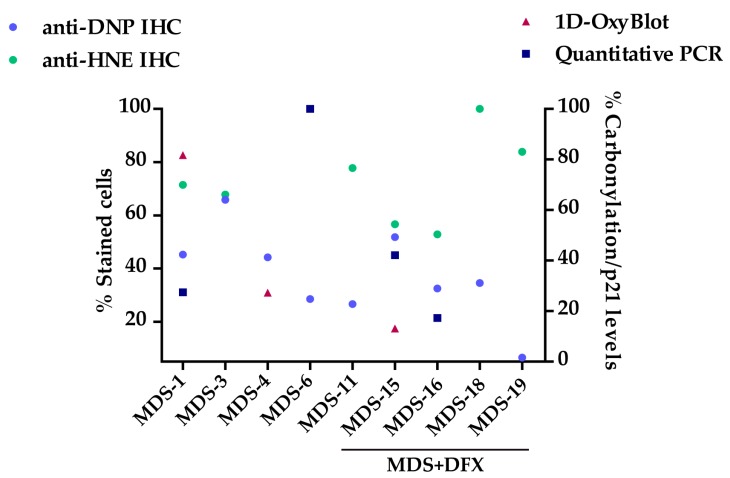
2D plot sample-by-sample of patients analyzed by several techniques. Correlation of the percentage of positive stained cells for DNP and HNE antibodies and the percentage of carbonylation/p21 levels from MDS patients (MDS-1, 3, 4, 6) and MDS patients treated with DFX (MDS-15, 16, 18, 19).

To study signaling pathways potentially activated by oxidative stress and involved in MDS pathogenesis [25], and their possible modulation by DFX, we analyzed the expression of the p53 target gene, p21, in BM samples. Results of qPCR analysis showed that the expression of p21 was considerably and significantly higher (*P* ≤ 0.001) in BM from MDS patients than from control samples (Figure 5a). Interestingly, p21 expression was modestly but significantly lower (*P* ≤ 0.05) in MDS samples after DFX treatment (Figure 5b), thus demonstrating an association between oxidative stress in MDS and p21 expression. Figure A4 summarizes the values obtained sample-by-sample when the same patients were analyzed by several techniques.

## 4. Discussion

Oxidative stress and its effects on cell biology, DNA damage and carcinogenesis are important in the pathogenesis of MDS [9,10]. In an attempt to identify possible effectors of oxidative damage, here we provide the first analysis, to our knowledge, of protein carbonylation in hematopoietic lineages from patients with MDS. This was, however, a challenging endeavor due to the limited number of patients with this pathology, the restricted amount of sample available for research and the difficulty in the expansion of cell progenitors. Analyzing BM samples by proteomics necessitates a large amount of sample, which likely explains the limited number of proteomic studies performed for this pathology.

Immunohistochemical analysis revealed an increase in protein carbonylation in BM samples from patients with MDS, mostly in cells of the myeloid series. This was probably not triggered by lipid peroxidation, as reflected in the similarities in 4-HNE staining between samples from patients and controls. As iron levels are elevated in MDS patients (Table 1), metal-catalyzed oxidation should be a major contributor to protein damage in myeloid cells. Proteomic analysis also revealed an increase in protein carbonylation in erythroid precursors, which could be related to the increased DNA damage in MDS CD34+ cells [4,41]. Indeed, a recent study demonstrated an increase in DNA damage in MDS patients, which was reverted by treatment with an iron chelator [6]. 

The present study demonstrates for the first time that protein carbonylation in BM samples from MDS patients is decreased by DFX treatment, pointing to an important role of iron overload and the potential therapeutic benefit of iron chelators, as has been described in patients with low-risk MDS [30].

Previous assessments of oxidative stress in BM of patients with MDS using antioxidant biomarkers showed that all BM cell types have an imbalance in ROS levels that is related to lower overall survival [6,10,42]. This phenotype was also reverted by iron chelation [6,42], suggesting that amelioration of the oxidative stress effects by DFX treatment is beneficial for these patients. Indeed, an improvement in the pathogenesis of MDS has previously been reported after DFX therapy [20,33]. 

Interestingly, the findings from 2D-OxyBlot analysis suggest that perhaps only a few proteins are responsible for the bulk of total protein carbonylation in MDS samples. Four carbonylated proteins were successfully identified by MS analysis and are discussed below.

Actin is extremely susceptible to carbonylation [43]. This process seems to occur at an extent of oxidative insult higher than that causing the oxidation of some critical amino acids residues and causes the disruption of the cytoskeleton. Indeed, the increase in carbonylated actin found in a number of medical conditions is associated with severe oxidative modifications leading to functional impairment [44]. Actin carbonylation also occurs in malignant transformation [43] and, accordingly, our data suggest that this modification could be a marker of MDS pathogenesis. 

Lactate dehydrogenase, a redox-active enzyme, reversibly converts pyruvate to lactate during anaerobic glycolysis [45]. Interestingly, in the context of oxidative stress, cells rely heavily on anaerobic glycolysis for ATP production [46]. Diseased cells upregulate anaerobic glycolytic enzymes – particularly LDH – to increase energy in the form of ATP to maintain homeostasis [47,48]. LDH oxidation results in a loss of its catalytic activity due to the modification of functional protein residues [47,49], and LDH carbonylation has been associated with decreased activity in disorders related to oxidative stress imbalance [50]. Our data suggest that oxidative damage to LDH observed in MDS could be a key switch for the cell dysplasia observed in this disease. 

The 14-3-3 proteins are ubiquitous proteins involved in myriad processes, including the regulation of metabolism, signal transduction, cell cycle control through the p53/p21 pathway [51], apoptosis, protein trafficking, transcription, stress responses, and malignant transformation [52]. They are known to regulate the activity of a broad array of targets via protein-protein interactions, modulating signaling pathways related to redox regulation [53,54]. In addition, cysteine residues in 14-3-3 proteins are redox sensors which, in the oxidized state, impact their biological activity [55]. These oxidized post-translational modifications in 14-3-3 proteins are known disease markers in atherosclerosis [56] and neurological disorders [52], and could also serve as indicators of MDS. 

Finally, ZNF846 is involved in transcriptional regulation through its association with DNA binding sites, to control the expression of multiple genes, and also acts as an RNA polymerase II transcription factor coadjutant by virtue of its sequence-specific DNA binding activity [57]. Oxidative modification of ZNF846, via zinc finger cysteine thiols, leads to the release of zinc molecules from the binding site. This results in the loss of zinc finger protein function related to DNA-binding and also in an increase of free zinc that may stimulate and interfere with cellular signaling cascades [58,59]. These processes could contribute to multiple cellular dysfunctions involved in the pathogenesis and progression of MDS. Therefore, further exploration of this marker as a potential key factor in MDS-associated diseases is warranted. Interestingly, a regulatory transcription factor-binding site for p53 in the ZNF846 gene promoter has been found in the GeneCards database (www.genecards.org).

Signaling pathways activated by oxidative stress can control the cell cycle via p53 [23]. As expected, we found that the mRNA expression of p21 was significantly increased in MDS patients with respect to controls, and was significantly reduced by DFX treatment. Thus, mechanistically, the amelioration of oxidative stress by DFX seems to involve, at least in part, the p53/p21 pathway, which is a major signaling pathway activated by oxidative stress and altered in MDS [24]. To our knowledge, this is the first study that demonstrates the mitigating effect of DFX on p21 expression in the context of MDS. Overall, our data suggest that treatment of MDS with DFX could be effective in (1) inhibiting/reversing protein carbonylation and its harmful downstream consequences and (2) restoring the altered signaling pathways associated with oxidative stress.

## 5. Conclusions

Patients with MDS present a higher level of protein carbonylation with respect to control peers, along with an altered p53/p21 signaling pathway. The identification of four main carbonylated proteins might suggest a role for these oxidation-sensitive proteins in the pathogenesis of MDS. Finally, our data on DFX treatment in patients indicates that the inhibition of protein carbonylation associated with enhanced p21 signaling is possible in MDS.

## Figures and Tables

**Table 1:** MDS patients’ characteristics.

Demographic Data			Clinical Features									Performed Analysis			
Patient (No)	Sex (M/F)	Age (years)	MDS Subtypes(WHO)	Hb (g/dL)	ANC (10^9^ /L)	PTLs (10^9^ /L)	Blasts (%)	Cytogenetics (FISH)	Risk Groups (IPSS)	Iron Chelation Therapy	Ferritin (ng/m L)	A	B	C	D
1	M	83	RCMD-RS	10.5	3	250	1	N	Low	No	779	✔	✔		✔
2	F	75	CMML	12.3	4.6	163	2	N	Low	No	45		✔		
3	M	84	RCMD	12.8	1.1	99	2	N	Low	No	88	✔			
4	M	59	RCMD-RS	9.4	1.5	177	1	N	Low	No	582	✔	✔	✔	
5	M	75	RAEB-1	14	1.7	164	6	N	Int-1	No	168	✔			
6	M	83	RAEB-1	11.4	1.2	80	9	Mon (7, 20). Del (5q, 12p. 17q)	Low	No	180	✔			✔
7	F	84	RCMD	11.4	2.9	144	4	N	Low	No	136		✔	✔	
8	M	81	RCMD-RS	9.9	3.2	363	1	N	Low	No	437		✔		
9	F	73	RAEB-2	11.5	3.4	440	15	N	Int-1	No	1123		✔		
10	F	90	RCMD	9.1	4.4	166	4	N	Int-2	No			✔		
11	F	87	RARS	8.8	4.2	306	1	N	Low	No	1547	✔			
12	F	70	RAEB-1	10.6	1	133	5	N	Low	No	24	✔			
13	M	82	RCMD-RS	9.9	3.2	363	1	N	Low	No	319	✔			
14	F	81	RARS	10.9	4.3	165	2	N	Low	No	157	✔			
15	M	66	RARS	10.3	4.4	414	2	N	Low	Yes	1137.6	✔	✔		✔
16	M	70	RARS	6.7	6	313	2	Del (20q)	Low	Yes	884	✔			✔
17	F	66	RAEB-2	10.2	9	161	16	Del (5q)	Int-1	Yes	163				✔
18	M	71	RCMD-RS	7.8	7.8	185	4	N	Int-1	Yes	643	✔			
19	M	71	RCMD	8.2	1.8	31	1	N	Low	Yes	206.2	✔			
20	M	57	RCMD	7.5	8	22	<1	N	Int-1	Yes	767	✔			
21	F	82	RAEB-1	11.3	8.6	26	7	Del (5q)	Int-1	Yes	1179	✔			

No = sample number; M = male; F = female; RCMD-RS = refractory cytopenia with multilineage dysplasia and ringed sideroblasts; CMML = chronic myelomonocytic leukemia; RCMD = refractory cytopenia with multilineage dysplasia; RAEB-1 = refractory anemia with excess of blasts type 1; RAEB-2 = refractory anemia with excess of blasts type 2; RARS = refractory anemia with ringed sideroblasts; Hb = hemoglobin; ANC = absolute neutrophil count; PTLs = platelets; IPSS =International Prognostic Scoring System; Low = low-risk; Int-1 = intermediate-1-risk; Int-2 = intermediate-2-risk; *N* = normal; Mon (7, 20) =, monosomy of chromosomes 7 and 20; Del(5q, 12p, 17q) = deletion of chromosome 5,12,7. Performed analyses methods: A = immunohistochemistry; B = one-dimensional OxyBlot; C = two-dimensional OxyBlot; D = quantitative polymerase chain reaction.

**Table 2:** Identification of carbonylated proteins.

Protein ID	Accesion No.	No. of Peptides Matched/Searched	%Coverage	%Score	Nominal Mass (Mr)/Pi
Actin, cytoplasmic 1	sp|P60709|ACTB_HUMAN	21/65	49	147	42,052/5.29
Zinc finger protein 846	sp|Q147U1|ZN846_HUMAN	12/65	26	68	62,109/9.21
14-3-3 protein zeta/delta	sp|P63104|1433Z_HUMAN	12/65	47	90	27,899/4.73
L-lactate dehydrogenase A chain	sp|P00338|LDHA_HUMAN	14/65	45	104	36,950/8.44

Protein ID = protein ID from NCBInr database; Accession no. = accession numbers from NCBInr database; No. of peptides matched/searched = number of matched peptides versus total number of peptides; %Coverage = coverage of the matched peptides in relation to the full-length sequence; %Score = probability-based MOWSE score; Nominal mass (Mr)/Pi = theoretical nominal mass (Mr) and isoelectric point (pI) from the NCBInr database.
